# Interleukin-1 Receptor Antagonist Modulates the Early Phase of Liver Regeneration after Partial Hepatectomy in Mice

**DOI:** 10.1371/journal.pone.0025442

**Published:** 2011-09-27

**Authors:** Antonino Sgroi, Carmen Gonelle-Gispert, Philippe Morel, Reto Marc Baertschiger, Nadja Niclauss, Gilles Mentha, Pietro Majno, Veronique Serre-Beinier, Leo Buhler

**Affiliations:** 1 Surgical Research Unit, Department of Surgery, Geneva University Hospital and University of Geneva, Geneva, Switzerland; 2 Visceral Surgery, Department of Surgery, Geneva University Hospital, Geneva, Switzerland; French National Centre for Scientific Research, France

## Abstract

**Background:**

Cytokine administration is a potential therapy for acute liver failure by reducing inflammatory responses and favour hepatocyte regeneration. The aim of this study was to evaluate the role of interleukin-1 receptor antagonist (IL-1ra) during liver regeneration and to study the effect of a recombinant human IL-1ra on liver regeneration.

**Methods:**

We performed 70%-hepatectomy in wild type (WT) mice, IL-1ra knock-out (KO) mice and in WT mice treated by anakinra. We analyzed liver regeneration at regular intervals by measuring the blood levels of cytokines, the hepatocyte proliferation by bromodeoxyuridin (BrdU) incorporation, proliferating cell nuclear antigen (PCNA) and Cyclin D1 expression. The effect of anakinra on hepatocyte proliferation was also tested in vitro using human hepatocytes.

**Results:**

At 24h and at 48h after hepatectomy, IL-1ra KO mice had significantly higher levels of pro-inflammatory cytokines (IL-6, IL-1β and MCP-1) and a reduced and delayed hepatocyte proliferation measured by BrdU incorporation, PCNA and Cyclin D1 protein levels, when compared to WT mice. IGFBP-1 and C/EBPβ expression was significantly decreased in IL-1ra KO compared to WT mice. WT mice treated with anakinra showed significantly decreased levels of IL-6 and significantly higher hepatocyte proliferation at 24h compared to untreated WT mice. *In vitro*, primary human hepatocytes treated with anakinra showed significantly higher proliferation at 24h compared to hepatocytes without treatment.

**Conclusion:**

IL1ra modulates the early phase of liver regeneration by decreasing the inflammatory stress and accelerating the entry of hepatocytes in proliferation. IL1ra might be a therapeutic target to improve hepatocyte proliferation.

## Introduction

Acute liver failure (ALF) occurs when the extent of hepatocyte death exceeds the liver's regenerative capacity. Furthermore, the regeneration of native liver may be impaired by accumulation of various toxic substances such as ammonia or nitric acid [1]. This clinical devastating syndrome is associated with a significant morbidity and mortality reaching 80%. Liver transplantation is currently the only effective therapy for those patients who are unlikely to recover with standard care. However, shortage of human organ donors limits the number of possible transplantations and additional approaches are crucial to reduce the waiting list [2].

The administration of specific drugs or factors that are able to initiate and accelerate hepatocyte proliferation has been suggested as strategy for the treatment of acute liver failure [3]. Thus, the improvement of liver regeneration by cytokine therapy is a potential solution to increase the number of patients in whom the native liver regenerates sufficiently to resume normal function.

Among the many cytokine candidates to improve liver regeneration, the cytokine IL-1 receptor antagonist (IL-1ra) has not yet been studied and direct evidence for a role of IL-1ra in liver regeneration has not been clearly reported [4], [5].

IL-1ra is a member of the IL-1 family and is produced by hepatocytes as an acute-phase protein [6], [7]. This cytokine has a naturally anti-inflammatory effect by binding and blocking competitively IL-1 receptor, i.e. preventing binding and intracellular signal transduction of IL-1α and IL-1β. The production of IL-1ra is upregulated by IL-1β, IL-4 and HGF. In the past few years, IL-1ra has attracted clinical attention because its serum levels are elevated in diverse human pathologies as infectious diseases, neoplasic diseases and liver diseases [8].

Recent studies have demonstrated that the activation of IL-1β signalling results in decrease of hepatocyte proliferation *in vitro* and *in vivo* and that its inhibition induces an improvement of mitogenic rate of hepatocyte during liver regeneration [9], [10], [11].

The plasma IL-1ra/IL-1 ratio in a healthy population is close to 1 and exhibits minimal variation [12]. Sekiyama et al. showed that in patients with fulminant hepatic failure a significantly reduced ratio of IL-1ra to IL-1 beta (IL-1ra/IL-1β) was observed in patients who subsequently died compared with subjects who survived [13]. In a rat model of fulminant hepatic failure induced by D-galactosamine, Shinoda et al. have shown that animal survival was significantly improved in animals treated with IL-1ra [14], [15]. Recently, overexpression or administration of IL-1ra in animal models has been shown to be protective in different liver injury such as hepatic ischemia-reperfusion injury and hepatitis [16], [17], [18].

Currently, a non glycosylated recombinant human IL-1ra (anakinra), is available for clinical use. As the endogenous IL-1ra, this drug blocks the effect of IL-1β and it is used to treat pain and swelling of patients with rheumatoid arthritis [19].

The aims of this study were first to evaluate the role of IL-1ra in liver regeneration *in vivo* using knock-out mice in which the gene coding for IL-1ra has been deleted and second to analyse the effect of anakinra (the non glycosylated recombinant human IL-1ra) administration on liver regeneration in wild type mice after 70%-hepatectomy and on isolated human hepatocytes *in vitro*.

## Materials and Methods

### Animals

10 to 12 week-old male wild-type (WT) DBA1 mice (Charles River Laboratories, France), and IL-1ra knock-out (KO) DBA1 mice weighing 20 to 25g were used. IL-1ra KO breeding was performed from animals previously described [20]. Animals were maintained in conventional housing facilities at the Geneva University Medical School. This study was conducted under experimental protocols approved by the ethical committee of the Geneva University Medical School and by Geneva veterinary authorities.

### Induction of liver regeneration

Regeneration of the native liver was induced by performing a 70%-hepatectomy. Briefly, under general anaesthesia, a median laparotomy was performed. The left lateral and the median lobe were removed as previously described by Mitchell et al. [21]. In sham operated mice, hepatectomy was not performed.

### Experimental groups

70%-hepatectomy was performed in four different groups of DBA1 mice:

- WT DBA1 mice, n = 6
- IL-1ra KO DBA1 mice n = 5
- WT DBA1 mice treated with intraperitoneal (i.p.) injection of anakinra (5mg/kg/day) (Kineret®, Amgen Europe B.V, Breda, Netherlands) n = 5
- WT DBA1 mice treated with i.p. injection of anakinra (50mg/kg/day) n = 5

Two groups contained sham operated DBA1 mice:

- WT DBA1 mice treated with i.p. injection of anakinra (5mg/kg/day) n = 5
- WT DBA1 mice treated with i.p. injection of anakinra (50mg/kg/day) n = 5

### Measurement of cytokines

Blood levels of IL-1ra, IL-1β, TNF-α and TGF-β1 (R&D system, Minneapolis, USA) and HGF (Institute of Immunology, Japan) were analyzed by enzyme-linked immunosorbent assay (ELISA), following the manufacturer's instructions. IL-6, MCP-1 were measured using a cytometric bead array (CBA) mouse inflammation kit (BD Biosciences, New Jersey, USA) and analyzed with a BD FACSArray Bioanalyzer (BD Biosciences), following the manufacturer's instructions. The CBA data were analyzed with BD™ CBA Software (BD Biosciences). The cytokines were measured from peripheral blood at 4 h, 24 h, 48 h, 72 h, 5 days and 7 days after hepatectomy.

### Measurement of alanine aminotransferase (ALT)

ALT was analyzed on peripheral blood of mice treated with anakinra (n = 3) and with no treatement (n  = 3) by the clinical chemistry unit of University Hospital Geneva using DxC 800 system (Beckman Coulter Inc), following the manufacturer's instructions.

### Bromodeoxyuridine (BrdU) incorporation

Hepatocyte proliferation was evaluated by Bromodeoxyuridine (BrdU) incorporation at 24 h, 48 h, 72 h, 5 days and 7 days after hepatectomy. BrdU (200mg/kg) was given i.p. 2 h before tissue sampling [22]. Immediately after sacrifice, liver samples were fixed in 10% buffered and formalin and paraffin embedded. Sections of 5 µm were cut. BrdU incorporation in hepatocyte nuclei was assessed by immunohistochemistry using Zymed BrdU STAINING Kit (Zymed Laboratories, San Francisco, CA, USA) following the manufacturer's instructions. The proliferation index of BrdU-stained tissue was determined at 200x magnification and labelled nuclei were counted in 5 randomly chosen fields, which approximate 1000 cells per section. Data were expressed as the percentage of BrdU-stained hepatocytes per total number of hepatocytes.

### Western blot analysis

Liver biopsies were removed at 4 h, 24 h, 48 h, 72 h, 5 days and 7 days after partial hepatectomy and frozen at −80°C until protein extracts were prepared by homogenization in a lysis buffer (0.1 M Tris-HCl buffer, pH 7.4, supplemented with 5 mM EDTA and 5% SDS) containing complete protease inhibitors cocktail (Roche, Basel, Switzerland) and phosphatase inhibitor sodium orthovanadate (1 µM). After a 20 min centrifugation at 14 000*g* at 4°C, the supernatant was collected, protein concentration of the protein extracts was determined using the Bio-Rad protein assay kit (Biorad, ville, pays) and finally samples were stored at -20°C until western blot analyses. 30 µg of total liver proteins were separated by electropohoresis in a 12% sodium dodecyl sulphate (Invitrogen, Taastrup, Denmark) polyacrylamide gel. Proteins were transferred onto polyvinylamide fluoride membranes (Hybond-P, GE Healthcare, Little Chalfont, United Kingdom). Membranes were blocked for 1 h at room temperature in a blocking buffer (Tris-HCl (pH 7.6) buffer containing 150 mmol/l NaCl, 0.1% Tween-20 and 5% non-fat dry milk). The membranes were then incubated overnight at 4°C with one of the following antibodies diluted in the blocking buffer: for PCNA, mouse monoclonal antibody clone: PC10 (Signet Laboratories, Inc, Dedham, MA, USA) diluted 1∶500; for Cyclin D1, mouse monoclonal antibody diluted 1∶500 (Santa Cruz Biotechnology, Inc., Heidelberg, Germany). After rinsing in TBS-Tween, the immunoblots were incubated for 1 h at room temperature with a goat anti-rabbit or anti-mouse secondary antibody (Hercules, CA, USA), whichever appropriate, conjugated to horseradish peroxidase and diluted 1∶6000 in the blocking buffer.

Finally, membranes were developed by enhanced chemiluminescence detection kit (Amersham Pharmacia Biotech, Piscataway, NJ) according to manufacturer's instructions. For all blots, amount of loaded proteins was controlled by probing the same membranes with a rabbit polyclonal antibody directed against β-actin diluted 1/250. Densitometric quantification of each band was determined using Quantity One software (PDI, Inc., Huntington Station, NY) and normalized by comparison with expression of β-actin in the re-probed blot.

### Analysis of Gene Expression by Real-time Polymerase Chain Reaction

Total RNA was extracted from liver samples harvested from WT DBA1 and IL-1ra KO DBA1 mice at 4 h, 24 h, 48 h, and 72 h after partial hepatectomy by Qiagen RNeasy Midi kit (Qiagen, San Diego, USA) according to manufacturer's instructions. cDNA was synthesized from 0.5 µg of total RNA using PrimeScript RT reagent Kit (Takara Bio Inc, Saint-Germain-en-Laye, France) following suppliers instructions.

For quantitative PCR, amplification of genes was performed from 2ng cDNA and 300 nM of forward and reverse oligonucleotides using the Power SYBR Green PCR Master Mix (Applied Biosystems Inc, California, USA) and a SDS 7900 HT machine (Applied Biosystems Inc). Oligonucleotides were obtained from Invitrogen. The efficiency of each design was tested with serial dilutions of cDNA. Oligonucleotides amplicons sequences are described in Table 1. PCR were performed with the following parameters: 50°C for two minutes, 95°C for ten minutes, and 45 cycles of 95°C 15 secondes−60°C one minute. Each reaction was performed in three replicates on 384-well plate. Raw Ct values obtained with SDS 2.2 (Applied Biosystems Inc) were imported in Excel and normalisation factor and fold changes were calculated using the GeNorm method [23]. Control genes used for normalization are rps9, eef1a1, Srp72, Gak. These genes were selected using Genorm method. Srp72 and Gak are the most stable genes in the liver according to genevestigator (https://www.genevestigator.com).

**Table 1:** Primers of genes implicated in liver regeneration.

Primers	Forward	Reverse
C/EBPα	5′ CCTGAGAGCTCCTTGGTCA-3′	5′-GAAACCATCCTCTGGGTC-TC-3′
C/EBPβ:	5′-ACGACTTCCTCTCCGACCT-3′	5′-GAGGCTCACGTAACCGTAGTC-3′
C-myc	5′-AGGCCCCCAAGGTAGTGATC-3′	5′-GTGCTCGTCTGCTTGAATGG-3′
Bclx	5′- CAGACACTGACCGTCCACTCA-3′	5′- GCAATGGTGGCTGAAGAGAGA-3′
PPARα	5′- TGCAAACTTGGACTTGAACG-3′	5′- AGGAGGACAGCATCGTGAAG-3′
IGFBP-1	5′- ATCTGCCAAACTGCAACAAG-3′	5′-GACCCAGGGATTTTCTTTC-3′

C/EBP α =  CAAT enhancer binding protein α, C/EBP β =  CAAT enhancer binding protein β, IGFBP-1 = Insulin-like growth factor binding protein 1, peroxisome proliferator-activated receptor-α.

### Isolation of Human Hepatocytes

Human hepatocytes were obtained from surgical liver biopsies of patients undergoing segmental hepatectomies. The protocol for the human studies was approved by the institutional ethics committee of the Department of Surgery and informed consent was obtained from the patients. At the start of the intervention, a wedge of macroscopically normal tissue (15 to 30 g) located within the part of the liver to be resected was excised, immersed in ice-cold phosphate buffered saline.

Human hepatocytes isolations were performed using a two-step collagenase perfusion method as previously described [24], [25]. Hepatocyte viability was determined by trypan blue exclusion and cells were cultured in DMEM/F12 medium (Invitrogen, Basel, Switzerland) containing 2% Foetal bovine serum (Invitrogen), 1×10^−6^ mol/l dexamethasone (Sigma-Aldrich GmbH, Basel, Switzerland), 1×10^−8^ mol/l 3, 3′-triiodo-L-thyronine, 1×10^−8^ mol/l human insulin (Huminsulin, Lilly France S.A.S, Strasbourg, France), 5 µg/ml apotransferrin (Sigma-Aldrich GmbH), 15×10^−3^ mol/l Hepes.

### *In vitro* studies

2×10^5^ primary human hepatocytes were seeded in 35mm Tissue Culture dish (Primaria Easy grip, Becton Dickinson, Le Pont de Claix, France) and incubated at 37°C for 3 days.

To analyze the effect of anakinra on primary human hepatocytes proliferation *in vitro*, we treated primary hepatocytes with anakinra (Kineret®) at various doses (10 µg/ml and 100 µg/ml) added to culture medium at 0h for 72h after hepatocyte isolation. After isolation, primary human hepatocytes were cultured for 24h before the beginning of treatment with anakinra.

After 24h, 48h and 72h, protein extracts were prepared by scrapping primary hepatocytes with lysis buffer (described above) containing complete protease inhibitors cocktail (Roche, Basel, Switzerland) and phosphatase inhibitor and stored at −20°C until assessment of PCNA protein level expression by western blot analysis.

### Cell viability

To study the cell viability, we performed a crystal violet assay. Human hepatocytes were first seeded at 100,000 cells/well in 500 µl culture medium in 24-well plates and allowed to attach for 24h before anakinra treatment. Cell viability was then determined by crystal violet staining (Sigma, St. Louis, MO). For this assay, human hepatocytes were seeded at a density of 100.000 cells/well in 24-well plates in medium containing 2% FBS, with or without adjunction of anakinra at 10 (n = 3) and 100 microgr/ml (n = 3) for 24h and viability was tested at 24, 48 and 72h.

After incubation, the cells were fixed with 10% formalin during 10 min. at room temperature and stained for 30 min. at 37°C with 0.1% crystal violet (Sigma). Then, wells were washed with PBS three times and the dye was eluted under shaking with 500 µl of 10% acetic acid for 15 min. at room temperature. Supernatants were transferred into a 96-well plates and absorbance of each well was measured at 570 nm using a microplate reader.

### Statistical analysis

Data analysis results are presented as means ± SEM. For *in vitro* studies, statistical differences among three or more groups were examined by Mann-Whitney U test. For *in vivo* studies, differences between two groups were examined for statistical significance using the Kruskal-Wallis Anova test. Quantitative variation was considered significant at p-value inferior to 0.05. Statistical analysis was performed using STATISTICA (STATISTICA 5.5 Software for Windows, Statsoft Inc, Tulsa, OK).

## Results

### Analysis of liver regeneration after a 70%-hepatectomy in WT and IL-1ra KO mice

#### Extent of liver regeneration

We analysed hepatocyte proliferation in native liver tissue of WT and IL-1ra KO mice after hepatectomy by immunohistochemistry on liver sections analyzing BrdU incorporation and by western blotting detecting PCNA expression at 24 h, 48 h, 72 h, 5 days and 7 days.

**Figure 1:**
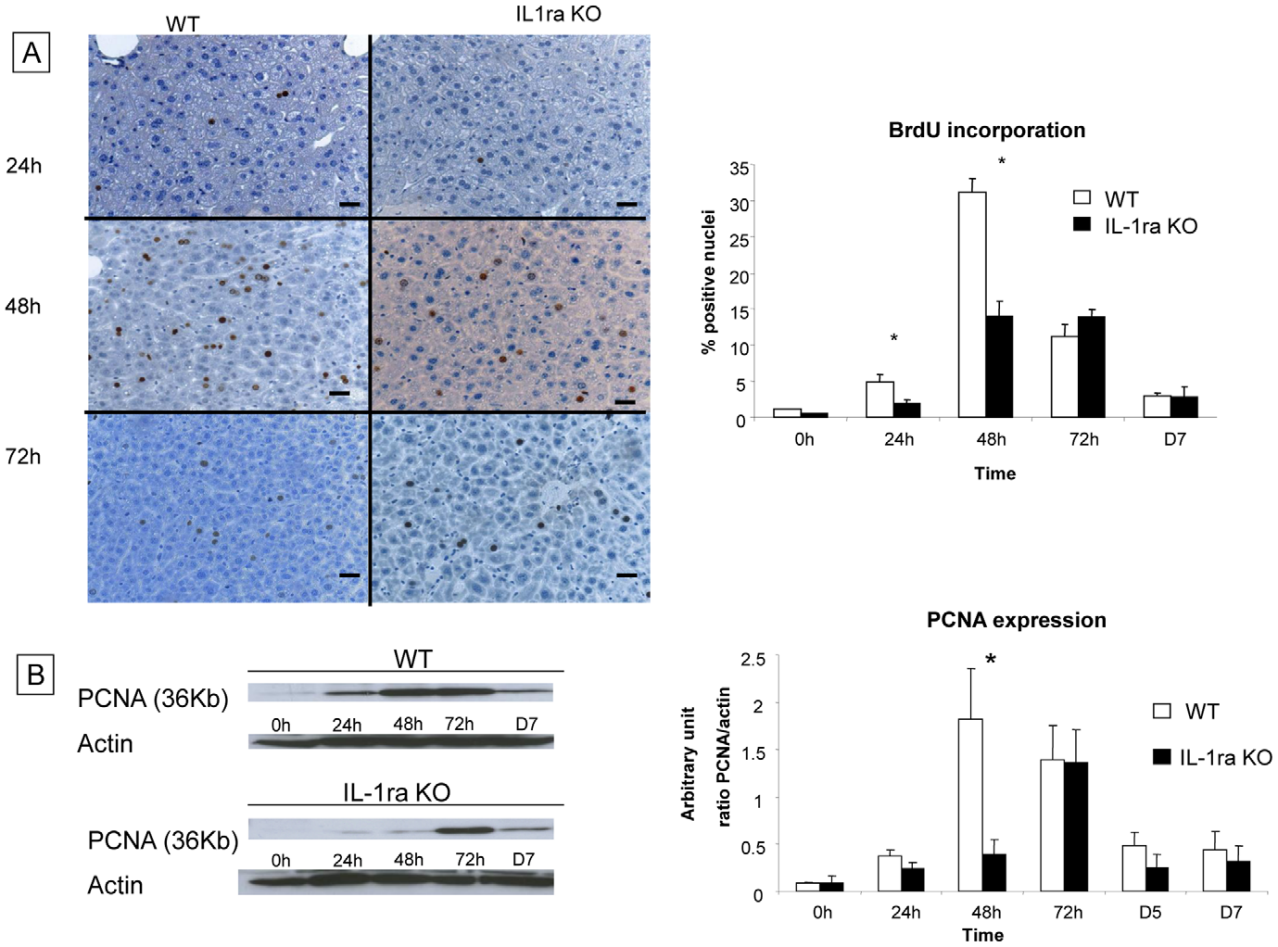
Evaluation of liver regeneration in IL-1ra ko mice and in WT mice after 70% hepatectomy. The evaluation of hepatocyte proliferation on native liver tissue was performed by analyzing BrdU incorporation by immunohistochemistry and by analyzing PCNA expression by western blot analysis at 24 h, 48 h, 72 h, 5 days and 7 days after 70% hepatectomy in WT mice and IL-1ra KO mice. (A) BrdU incorporation was significantly higher in WT mice compared to IL-1ra KO mice at 24 h and 48 h after 70% hepatectomy. The proliferation index of BrdU-stained tissue was determined at 200x magnification and labelled nuclei were counted in 5 randomly chosen fields, which approximate 1000 cells per section. Data were expressed as the percentage of BrdU-stained hepatocytes per total number of hepatocytes. Positive hepatocytes for BrdU incorporation are stained in brown. (Original magnification x200). (B) For WT mice, PCNA expression peaked at 48 h, in contrast for IL-1ra KO mice, the PCNA expression peaked at 72 h. The results are shown as a ratio of PCNA to actin expression. The quantification of signals of PCNA and actin are performed by densitometry. Scale bars  =  50 µm. *  =  Statistical significance p<0.05. D = day, WT = wild type, KO = knock-out, PCNA = proliferating cell nuclear antigen.

As shown in figure 1A, BrdU incorporation was increased in both WT and IL-1ra KO mice at 24 h after hepatectomy and reached maximal levels at 48 h. BrdU incorporation was significantly lower in IL-1ra KO mice compared to WT mice at 24 h (2% vs 5%, respectively) and 48 h (14% vs 32%, respectively) after 70% hepatectomy and return to similar levels at 72 h, and 7 days.

Western blot analysing PCNA expression (Figure 1B) increased at 24 h after hepatectomy in WT and in IL-1ra KO mice but was also significantly lower in IL-1ra KO mice at 48 h compared to WT mice (1.82 arbitrary unit (a. u.) ±0.53 vs 0.40±0.33, respectively) confirming the results obtained by BrdU incorporation. Maximal PCNA expression (1.82 a.u.) was observed in WT mice 48 h after hepatectomy while, in IL-1ra KO mice, maximal PCNA expression (1.33 a.u.) was measured 72 h after hepatectomy (Figure 1B).

#### Cytokines levels implicated in liver regeneration

**Figure 2:**
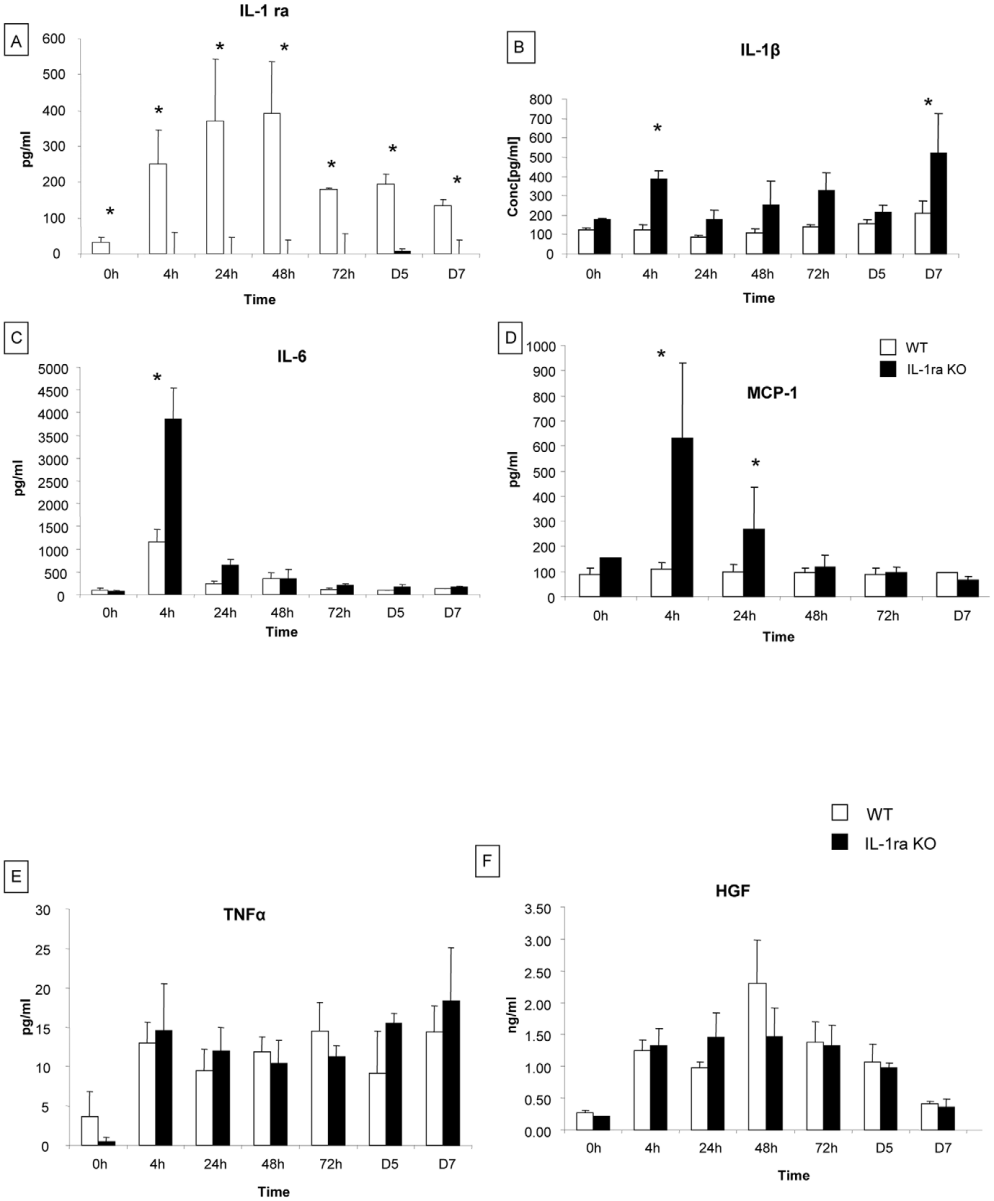
Cytokine profile during liver regeneration in WT mice and in IL-1ra ko mice after hepatectomy. IL-1ra, IL-1β, TNFα and HGF secretion were analyzed by ELISA. IL-6, MCP-1 were measured by a cytometric bead array mouse inflammation kit. These cytokines were measured on peripheral blood of WT mice and IL-1ra KO mice at 4 h, 24 h, 48 h, 72h, 5 days and 7 days after partial hepatectomy. A) The level of IL-1ra in WT mice showed a significant increase between 4 h and 48 h. As expected, in IL-1ra KO mice, IL-1ra was not detectable; B-D) the levels of IL-1β, IL-6 and MCP-1 were significantly higher in IL-1ra KO mice than in WT mice with a significant difference at 4 h post-hepatectomy; E-F) there were no differences in the levels of TNFα and HGF between the two groups of mice; *  =  Statistical significance p<0.05. D  =  day IL-1ra  =  Interleukin-1 receptor antagonist, IL-1β  =  Interleukin-1β, IL-6  =  Interleukin-6, MCP-1  =  monocyte chemotactic protein-1, TNFα  =  Tumor necrosis factor- α, HGF  =  Hepatocyte growth factor, WT  =  wild type, KO  =  knock-out.

IL-1ra, IL-1β, TNF-α, TGF-β1, HGF, IL-6 and MCP-1 levels were measured in serum of WT and IL-1ra KO mice at 4 h, 24 h, 48 h, 72 h, 5 days and 7 days after partial hepatectomy. In WT mice, IL-1ra level increased significantly between 4 and 48 h after hepatectomy (33±12 pg/ml at 0 h, 252±92 pg/ml at 4 h, 390±147 pg/ml at 48 h) (Figure 2A). After 48 h, the IL-1ra level progressively decreased until 7 days but was still remained higher than before hepatectomy. As expected, IL-1ra KO mice did not produce IL-1ra (Figure 2A). IL-1β levels were higher in IL-1ra KO than in WT mice at all time points with significant differences at 4 h (383±44 pg/ml vs 125±26 pg/ml, respectively) and at 7 days (328±89 pg/ml vs 140±9 pg/ml, respectively) after hepatectomy (Figure 2B). IL-6 level increased at 4 h post-hepatectomy in WT and IL-1ra KO mice. This increased secretion was significantly higher in IL-1ra KO than in WT mice (1164±273 pg/ml vs 3862±680 pg/ml, respectively) (Figure 2C). A significant increase of MCP-1 level was observed in IL-1ra KO mice at 4 h and at 24 h versus WT mice (629.9±109 pg/ml vs 109±27 pg/ml at 4h and 268±69.2 pg/ml vs 24.78 respectively) after hepatectomy (Figure 2D). TNF-α and HGF levels increased 4 h after hepatectomy in WT and IL-1ra KO mice (Figure 2E-F). There were no differences in the levels of TNF-α and HGF between the two groups of mice. These data suggest that IL-1 signalling pathways may regulate the expression of IL-6 and MCP-1 in the early phase of liver regeneration after hepatectomy.

#### Analysis of cell cycle protein expression

To determine whether the delay and decrease of hepatocyte proliferation observed in IL-1ra KO mice could be a consequence of alterations in the G1/S checkpoint of the cell cycle, cyclin D1 expression was analyzed by western blotting at 24 h, 48 h, 72 h and 7 days after 70% hepatectomy in WT and IL-1ra KO mice. Cyclin D1 was recently identified as the most reliable marker for cell cycle (G_1_ phase) in hepatocytes [26].

**Figure 3:**
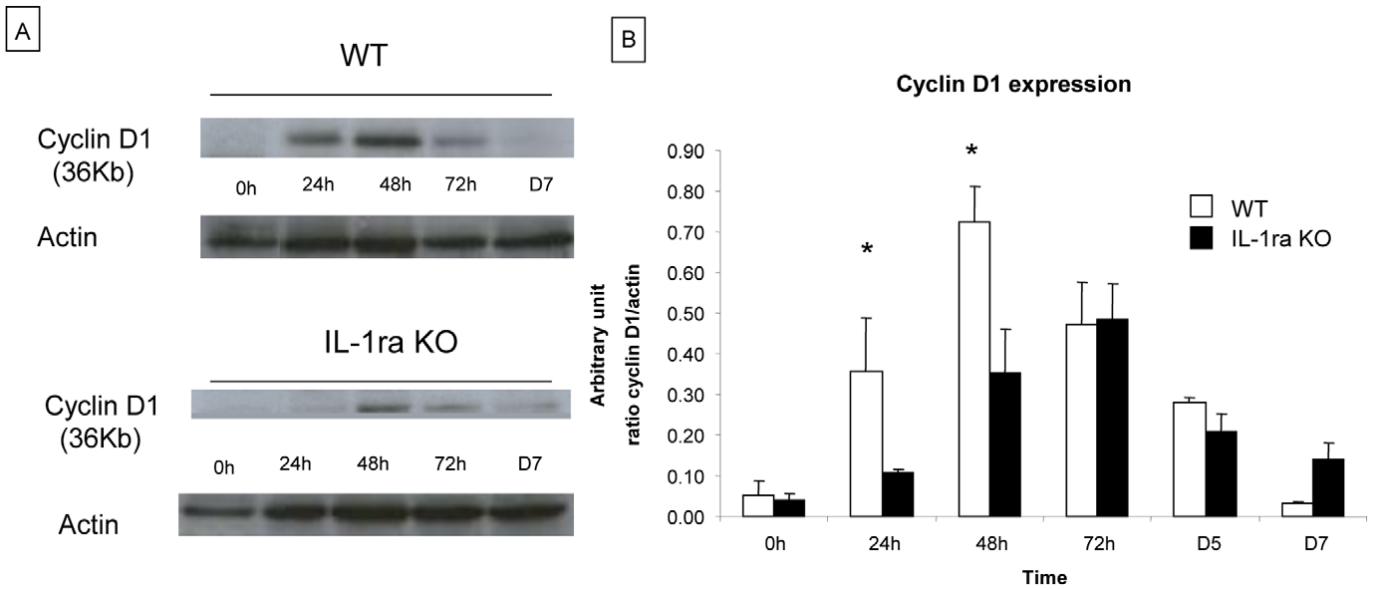
Analysis of Cyclin D1 expression in IL-1ra KO mice and in WT at early time points after hepatectomy. Expression of cell cycle protein Cyclin D1 was analysed by western blot analysis on native liver tissue recovered at 24 h, 48 h, 72 h, 5 days and 7 days after 70% hepatectomy in WT and IL-1ra KO mice. A) Western blot of cyclin D1. The quantification of bands of Cyclin D1 and actin are performed by densitometry. B) Cyclin D1 is significantly higher in WT mice compared to IL-1ra KO mice at 24 h and 48 h after 70% hepatectomy. Cyclin D1 increased significantly at 24 h in WT mice, and in IL-1ra KO mice at 48 h after hepatectomy. The results are expressed as a ratio of Cyclin D1 expression by actin expression. The quantification of bands of Cyclin D1 and actin are performed by densitometry. *  =  Statistical significance p<0.05. D  =  day, WT  =  wild type, KO  =  knock-out .

As shown in figure 3A and 3B, Cyclin D1 expression increased significantly between 0 to 48 h after 70% hepatectomy in WT mice (0.05±0.4 a.u. at 0 h, 0.36±0.13 a.u. at 24 h, 0.72±0.09 a.u. at 48h and 0.47±0.2 a.u. at 72h). In IL-1ra KO mice, a significant increase of expression was observed later, between 24 and 72 h (0.04±0.02 a.u. at 0 h, 0.11±0.01a.u. at 24 h, 0.35±0.11 a.u. at 48 h and 0.48 a.u. at 72 h). These results indicate a delayed cyclin D1 expression in IL-1 ra KO mice after partial hepatectomy. These results indicate a delayed cyclin D1 expression in IL-1 ra KO mice after partial hepatectomy.

#### Analysis of genes implicated in liver regeneration

**Figure 4:**
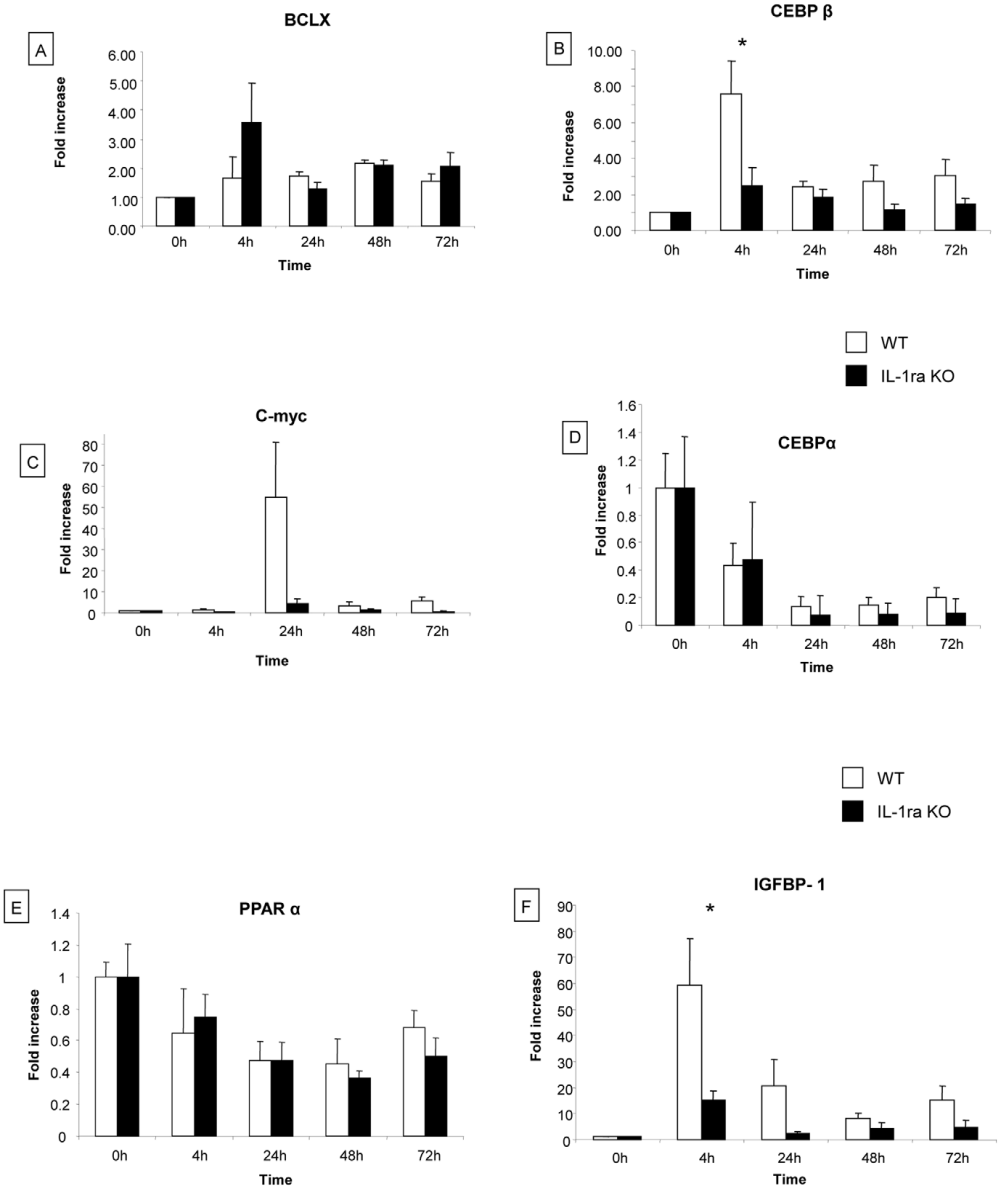
Expression of genes involved in liver regeneration. We analysed the expression of various genes implicated in liver regeneration (Bcl-xl, C/EBPα, C/EBPβ, c-myc, IGFBP-1 and PPARα) by real-time polymerase chain reaction at various time points 0 h, 4 h, 24 h, 48 h, 72 h post-hepatectomy. (A–H) Our results demonstrate that IGFBP-1 and C/EBPβ expression was significantly increased at 4 h in WT mice compared to IL-1ra KO (fold increase 59.1 vs. 15.3 for IGFBP-1 and 7.5 vs. 2.48 for C/EBPβ, respectively). Bcl-xl and c-myc expression was higher in WT mice but did not reach statistical significance. Expression level of C/EBPα and PPARα was similar. The fold increase was calculated for various time points and time 0 (0h) in control animals was used for normalization. *  =  Statistical significance p<0.05. WT  =  wild type, KO = knock-out, C/EBPα  =  CAAT enhancer binding protein α, C/EBPβ  =  CAAT enhancer binding protein β, IGFBP-1 =  Insulin-like growth factor binding protein 1, PPARα  =  Peroxisome proliferator-activated receptor alpha.

Expression of genes implicated in liver regeneration (Bcl-xl, CEBPα, C/EBP β, c-myc, IGFBP-1 and PPARα) was analyzed by real-time polymerase chain reaction at 0 h, 4 h, 24 h, 48 h, and 72 h after partial hepatectomy (Figure 4).

Expression levels of Bcl-xl, C-myc and PPARα were not modified in WT and IL-1ra KO mice (Figure 4 A, C, E). The expression of Bcl-xl at 4 h and c-myc at 24 h tended to be lower in IL-1ra KO mice compared to WT mice, but the difference did not reach statistical significance (Figure 4 A and C). Expression of C/EBPα decreased after partial hepatectomy in a similar way for IL-1ra KO and WT mice (Figure 4D). IGFBP-1 and C/EBPβ expression increased at 4 h after hepatectomy and were significantly lower in IL-1ra KO mice compared to WT mice (fold increase 15.3 vs 59.1. for IGFBP-1 and 2.48 vs 7.5. for C/EBPβ, respectively) (Figure 4B and 4F).

### Analysis of liver regeneration after 70% hepatectomy in WT mice treated by anakinra

In order to investigate whether the treatment of anakinra has a positive effect on hepatocyte proliferation after injury, we performed a 70% hepatectomy in WT mice treated with anakinra at two doses (5mg/kg/day and 50mg/kg/day).

#### Extent of liver regeneration

**Figure 5:**
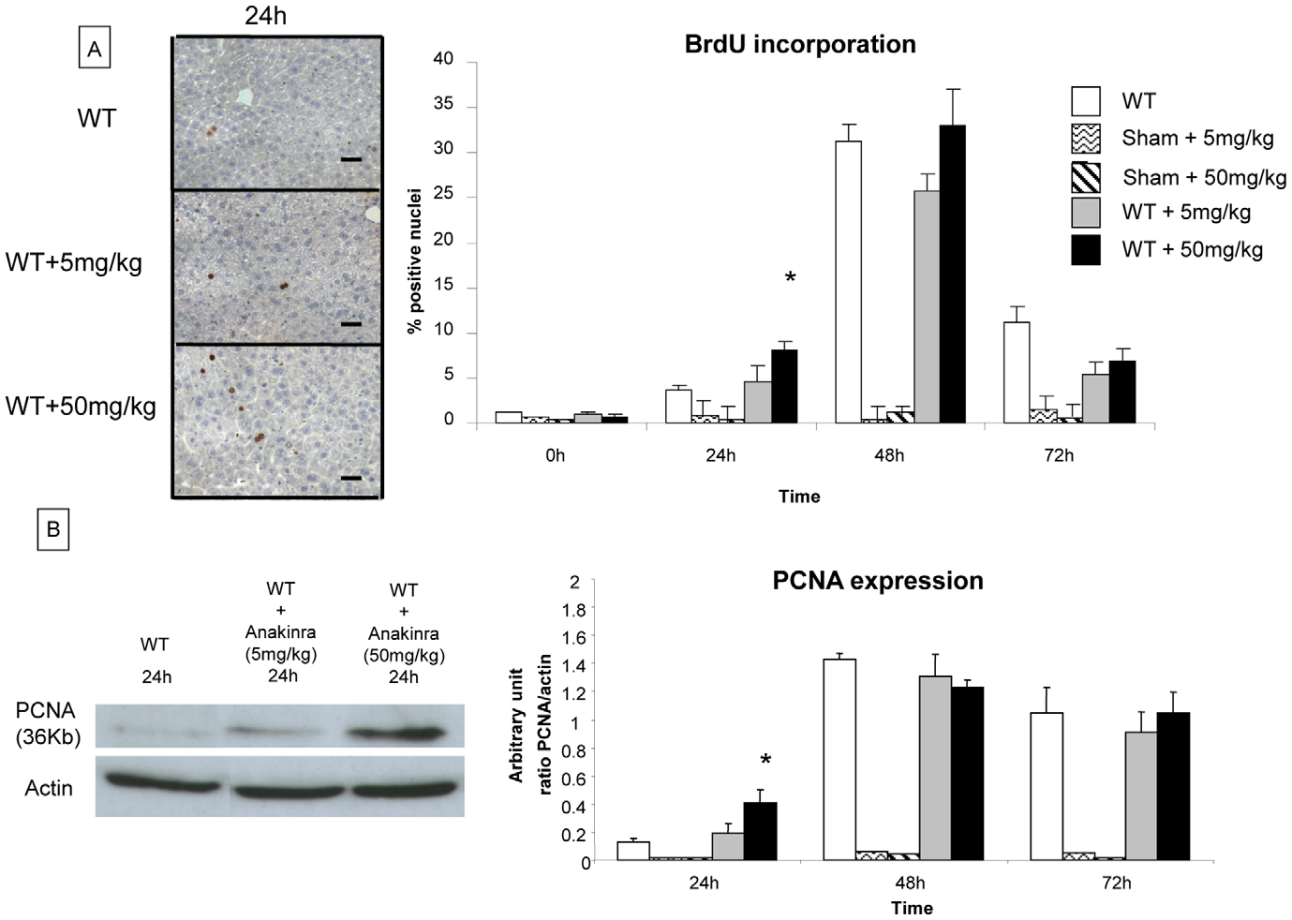
Evaluation of liver regeneration in mice treated by anakinra at 5mg/kg/d and at 50mg/kg/d after 70% hepatectomy. The evaluation of hepatocyte proliferation on native liver tissue was performed by analyzing BrdU incorporation by immunohistochemistry and by analyzing PCNA expression by western blot analysis at 24 h, 48 h, 72 h, 5 days and 7 days after 70% hepatectomy in untreated WT mice and groups of WT mice treated with anakinra (5mg/kg/d and 50mg/kg/d for 4 days). (A) BrdU incorporation was significantly higher in WT mice treated with anakinra at 50mg/kg compared to untreated mice at 24 h. At a 10 times lower dose (5mg/kg/d), BRDU incorporation was not different at 24 h in treated and untreated WT mice. At 48 h, the PCNA expression level was similar in all three groups of mice. The proliferation index of BrdU-stained tissue was determined at 200x magnification and labelled nuclei were counted in 5 randomly chosen fields, which approximately 1000 cells per section. Data were expressed as the percentage of BrdU-stained hepatocytes per total number of hepatocytes. Positive hepatocytes for BrdU incorporation are stained in brown. (Original magnification x200) (B) PCNA expression was significantly higher in WT mice treated with anakinra at a dose of 50mg/kg/d compared to untreated WT mice at 24 h after 70% hepatectomy. At a 10 times lower dose (5mg/kg/d), PCNA expression was not different at 24 h in treated and untreated WT mice. At 48 h, the PCNA expression level was similar in all three groups of mice. Scale bars  =  50 µm. *  =  Statistical significance p<0.05. WT = wild type, PCNA = proliferating cell nuclear antigen, BrdU = bromodeoxyuridine.

Hepatocytes proliferation from untreated and anakinra treated (5mg/kg/d and 50mg/kg/d for 4 days) WT mice was evaluated by BrdU incorporation (analyzed by immunohistochemistry) and by PCNA expression (analyzed by western blotting) at 24 h, 48 h, 72 h, 5 days and 7 days after 70% hepatectomy. BrdU incorporation was significantly higher in WT mice treated with anakinra at 50mg/kg compared to untreated mice at 24 h (8.04% vs 3.63% at 24 h, respectively) (Figure 5). BrdU incorporation was not different between the untreated and anakinra-treated mice at later time points (48 h and 72 h) after partial hepatectomy. As a control, the treatment of anakinra alone had no effect on BrdU incorporation in sham-operated mice (Figure 5A).

Similary, PCNA expression level was significantly higher in WT mice treated with anakinra at 50mg/kg/d compared to untreated WT mice at 24 h after 70% hepatectomy (0.40±0.09 a.u. vs 0.12±0.02 a.u., respectively) (Figure 5). At the same time point, treatment of mice with a lower dose of anakinra (5mg/kg/d) had no effect on the PCNA expression level. At 48 h and 72 h after hepatectomy, PCNA expression was similar between untreated and treated mice at low and high doses of anakinra. Finally, the treatment of anakinra alone had no effect on PCNA expression in sham-operated mice (Figure 5B). These data show that treatment with non-glycosylated recombinant human IL-1ra, anakinra at a dosis of 50 mg/kg/d, leads to an increased hepatocyte proliferation 24 h after partial hepatectomy.

#### Cytokines levels implicated in liver regeneration

**Figure 6:**
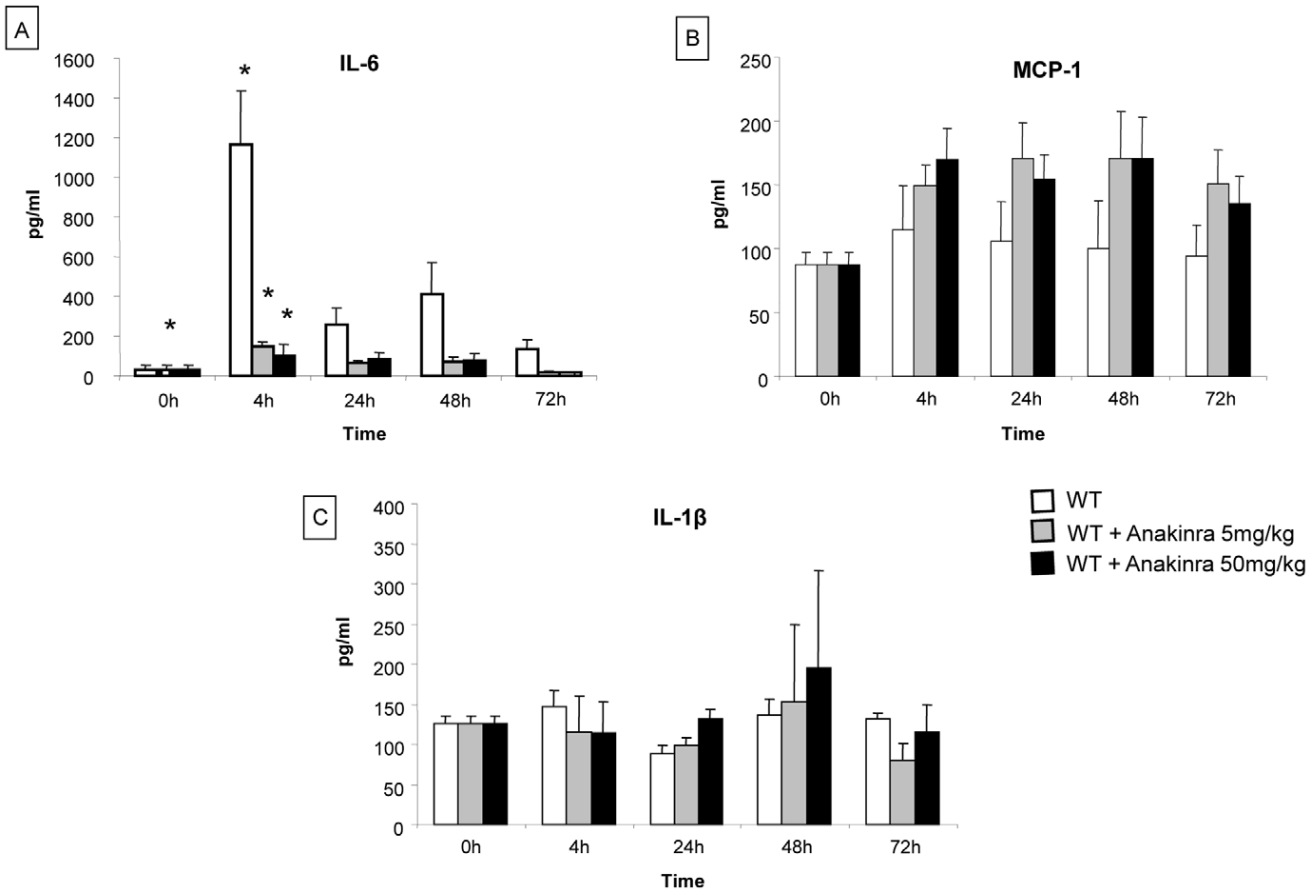
Cytokine profile during liver regeneration in WT mice treated by anakinra after hepatectomy. The cytokines IL-6, MCP-1 and IL-1β were measured on peripheral blood of WT mice untreated or treated with two doses of anakinra (5mg/kg/d and 50mg/kg/d for 4 days) at 4 h, 24 h, 48 h and 72 h after partial hepatectomy. A) At 4 h post-hepatectomy, IL-6 levels increased significantly in the three groups of mice compared to levels before hepatectomy. However, the levels of IL-6 were significantly higher in untreated mice than in WT mice treated with anakinra at 4 h post-hepatectomy. However there was no difference between mice treated with 5mg/kg and those treated with 50mg/kg; B) the levels of MCP-1 were higher but did not reach statistical significance in mice treated with anakinra compared to untreated mice at 4 h post-hepatectomy; C) There was no difference in the levels of IL-1β between the three groups of mice. *  =  Statistical significance p<0.05. IL-6  =  Interleukin-6, MCP-1  =  monocyte chemotactic protein-1, IL-1β  =  Interleukin-1β, WT  =  wild type.

IL-6, MCP-1 and IL-1β levels were measured from peripheral blood of WT mice either untreated or treated with two doses of anakinra (5mg/kg/d and 50mg/kg/d for 4 days) at 4 h, 24 h, 48 h, and 72 h, after partial hepatectomy. At 4 h post-hepatectomy, IL-6 levels were significantly reduced in WT mice treated with low and high doses of anakinra compared to untreated WT mice (149±16 pg/ml, 169±24 pg/ml vs 1164±273 pg/ml respectively treated with 5mg/kg/d and 50mg/kg/d anakinra vs untreated mice) (Figure 6A). Although MCP-1 levels tended to be higher in mice treated with anakinra compared to untreated mice at 4 h post-hepatectomy (Figure 6B), this difference was not statistically significant. Finally, IL-1β levels were similar between the untreated and treated mice (Figure 6C). These data show that treatment with anakinra leads to a decreased secretion of the pro-inflammatory cytokine IL-6 early after partial hepatectomy.

#### Measurement of alanine aminotransferase (ALT)

**Figure 7:**
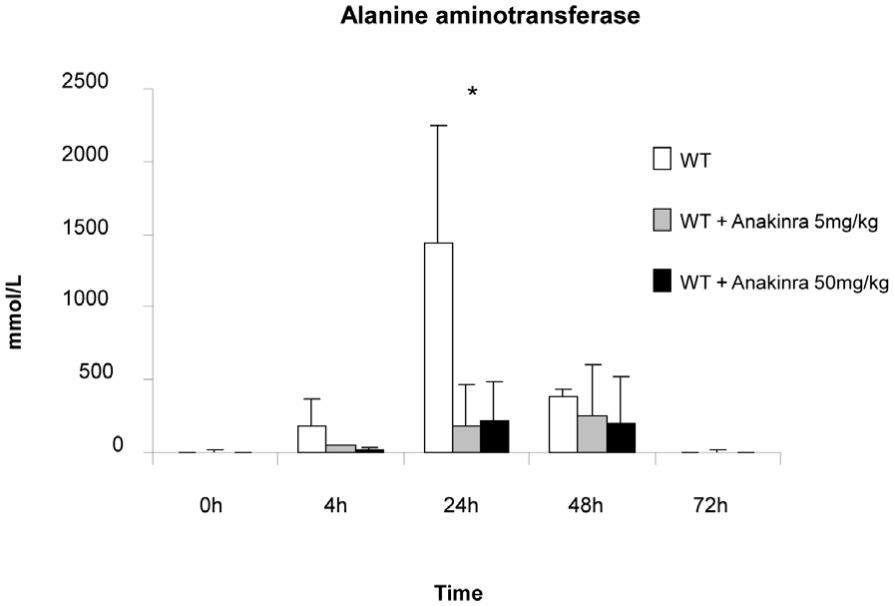
Measurement of alanine aminotransferase. Alanine aminotransferase (ALT) was analyzed on peripheral blood of WT mice treated with anakinra (n = 3) compared to WT mice without treatment (n = 3). Our results showed that alanine aminotransferase increase at 4h after partial hepatectomy and returned to normal levels after 72h. The levels of alanine aminotransferase of WT mice without treatment was significantly higher at 24h after partial hepatectomy compared to mice treated with anakinra (5 or 50mg/kg). For treated mice, there was no difference for the protective effect of anakinra between 5 and 50mg/kg. *  =  Statistical significance p<0.05. WT  =  wild type.

ALT was analyzed on peripheral blood of WT mice treated with anakinra (n = 3) and compared to WT mice without treatment (n = 3). Our results showed that alanine aminotransferase increase at 4h after partial hepatectomy and returned to normal levels after 72h. The levels of alanine aminotransferase of WT mice without treatment was significantly higher at 24h after partial hepatectomy compared to mice treated with anakinra (5 or 50mg/kg). For treated mice, there was no difference for the protective effect of anakinra between 5 and 50mg/kg (Figure 7).

### Effect of anakinra on the proliferation of primary human hepatocytes

**Figure 8:**
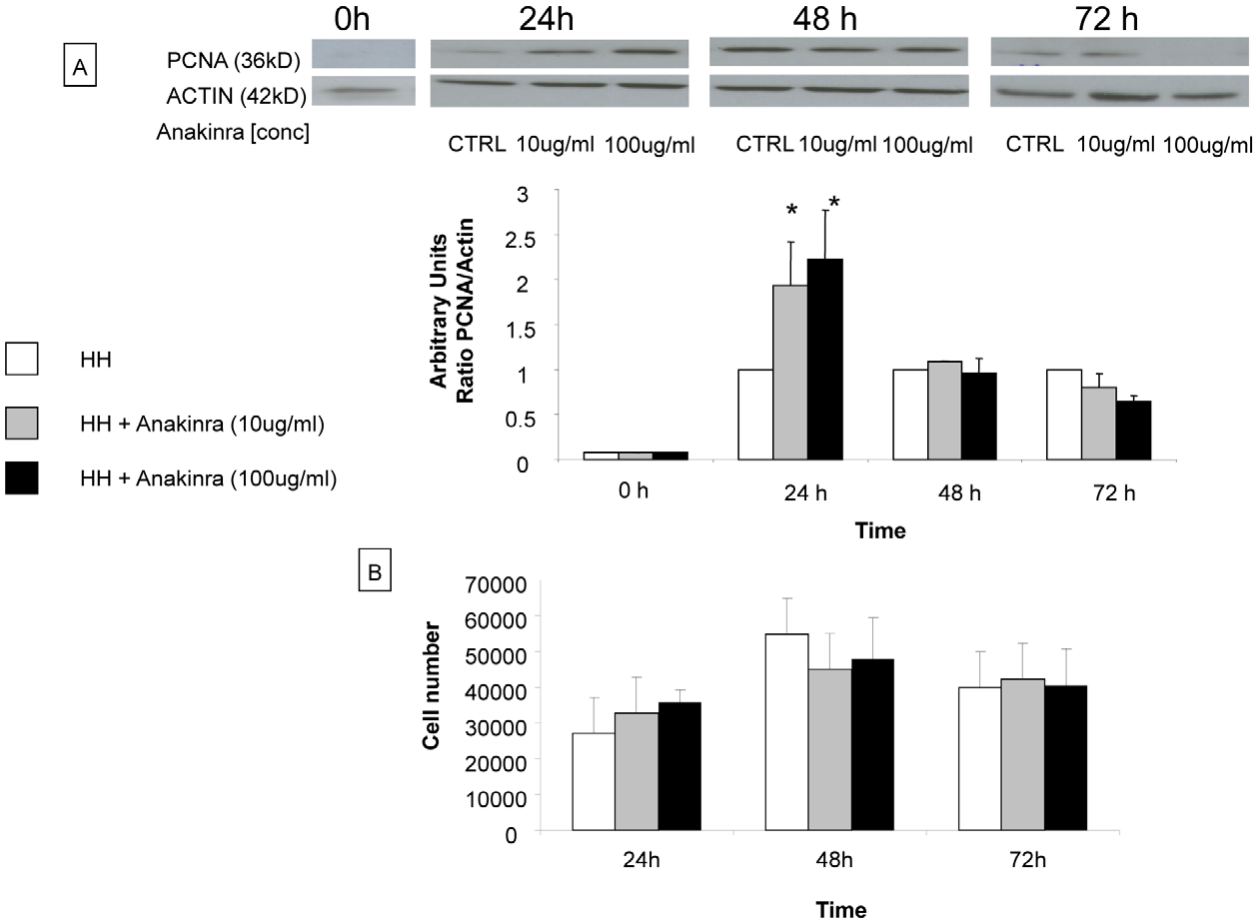
Evaluation of proliferation of primary human hepatocytes treated by anakinra in vitro studies. A) Primary human hepatocytes were isolated and cultured for 24 h before treatment with anakinra (10 µg/ml and 100 µg/ml). After 24 h, 48 h and 72 h proteins were extracted and whole cell lysats were subjected to western blotting. At 24 h, PCNA expression was significantly increased in hepatocytes treated with anakinra compared to non-treated hepatocytes. This difference is not observed at the other time points. The results are expressed as a ratio of PCNA expression by actin expression and are normalized in relation to none treated human hepatocytes. The quantification of bands of PCNA and actin were performed by densitometry. B) To study the cell viability, we performed a crystal violet assay. Human hepatocytes were first seeded at 100,000 cells/well in 500 µl culture medium in 24-well plates with or without adjunction of anakinra at 10 and 100 microgr/ml for 24h and viability was tested at 24, 48 and 72h. Our results showed that the cell viability was similar for all culture conditions (with or without anakinra) at various time points (n = 3). *  =  Statistical significance p<0.05. HH  =  primary human hepatocyte, PCNA  =  proliferating cell nuclear antigen.

*In vitro*, proliferation of primary human hepatocytes was evaluated by analyzing PCNA expression by western blotting at 24 h, 48 h and 72 h after treatment with anakinra (10 µg/ml and 100 µg/ml). PCNA expression was significantly higher in primary human hepatocytes treated with anakinra at 10 µg/ml and at 100 µg/ml in the first 24 h after treatment compared to primary human hepatocytes without treatment (fold increase 2.0±0.4, 2.2±0.5 vs 1.0, respectively) (Figure 8A). After 48 h and 72 h of anakinra treatment, PCNA expression level was similar between untreated and treated human hepatocytes (Figure 8A). In Figure 8B, our results showed that the cell viability was similar for all culture conditions (n = 3) (with or without anakinra) at various time points.

## Discussion

In the present study, we characterized the effects of *IL-1ra* gene disruption and IL-1ra treatment on liver regeneration of mice after partial hepatectomy. IL-1ra deficiency lead to a delay and a decrease in liver regeneration analyzed by BrdU incorporation and PCNA expression compared to WT mice. Peak of hepatocyte proliferation was observed at 72 h post-hepatectomy in IL-1ra KO mice compared to 48 h post-hepatectomy in WT mice and then decreased until reaching basal level at 7 days after hepatectomy.

Studying cytokine secretions after partial hepatectomy, we observed that IL-1ra KO mice demonstrated higher serum levels of IL-1β, IL-6 at 4 h and MCP-1 levels at 4 h and 24 h (in the proliferation phase) compared to WT mice. Indeed, as shown by several authors using various cell types including hepatic cells, IL-1ra blocks the production of IL-1β, IL-6 and MCP-1 [14], [15], [27], [28]. Increased MCP-1 secretion could also be related to the increased levels of IL-1β observed in IL-1ra KO mice as this cytokine has been reported to increase MCP-1 synthesis in non hepatic cells [29], [30] and stellate cells [31], [32].

These increased levels of pro-inflammatory cytokines may explain the delayed and reduced liver regeneration observed in IL-1ra KO mice. Indeed, IL-1β has been previously shown to antagonize hepatocyte proliferation [9], [10], [11]. Moreover, while IL-6 is known to stimulate the priming phase of liver regeneration, previous studies have shown that an overdose of IL-6 inhibits liver regeneration and delays cell cycle progression after partial hepatectomy [33], [34], [35], [36]. Finally, CC chemokine family members such as MCP-1 have been shown to modulate liver inflammation [37] and to play a role in the ischaemia/perfusion injury [38]. Although the mechanism of action of MCP-1 in the liver inflammatory process is unclear, this cytokine may directly cause impairment of hepatocyte proliferation. It is particularly noteworthy that MCP-1 serum levels were highest in patients with fulminant hepatic failure and fatal outcome compared to patients with acute non-fulminant hepatitis and favourable outcome [39].

The next phase of liver regeneration includes activation of tyrosine kinase receptor, c-met and epidermal growth factor (EGF) ligands, which, in turn, activate expression of transcription factors involved in liver regeneration. The main transcription factors activated after partial hepatectomy are c-jun, C/EBPβ and cAMP-responsive element modulator (CREM) [40]. Studies in C/EBP-β knockout mice revealed that this transcription factor was required for both gluconeogenesis and cell proliferation during liver regeneration [41]. As expected, C/EBP-β expression was significantly increased in the early phase of liver regeneration in WT mice and remained unchanged in IL-1ra KO mice after partial hepatectomy suggesting that the transcription factor C/EBP- β is modulated via IL-1ra.

Another important liver-specific immediate-early gene activated during liver regeneration and implicated in the maintenance of hepatocyte differentiation is the insulin-like growth factor binding protein 1 (IGFBP-1) [42]. The role of IGFBP-1 in liver regeneration is unknown but IGFBP-1 knockout mice showed a reduced and delayed hepatocyte DNA replication after partial hepatectomy [43]. While IGFBP-1 expression was significantly increased in the early phase of liver regeneration in WT mice, a very mild activation of IGFBP-1 gene was observed for IL-1ra KO. Several studies showed a link between IGFBP-1, C/EBP-β expression and IL-1 signalling pathway [43]. Thus, the decrease of IGFBP-1 and C/EBP-β expression in IL-1ra KO may explain the reduction and the delay of liver regeneration.

During liver regeneration, transition through S-phase requires the synthesis of a group of proteins such as DNA polymerase α, c-myc and cdc2 [44]. We observed that expression of early and delayed genes and transcription factors were also altered in IL-1ra KO mice after partial hepatectomy. Expression of the immediate early C-MYC gene was not statistically different between WT and IL-1ra KO mice, although C-MYC expression tended to be lower in IL-1ra deficient mice. The lower increase in expression of transcription factors and immediate early genes described above may alter the hepatocyte cell cycle and delay the transition through the S-phase.

Further progress through the cell cycle is dependent on activation by growth factors including HGF. During the priming phase (from 0 h to 20 h after hepatectomy) and proliferation phase (from 20 h to 5 days), we observed a significant increase in IL-1ra level and HGF in WT mice. These increased levels were observed until 48 h post-hepatectomy (proliferation phase) and then progressively decreased. These results are in accordance with Molnar et al. demonstrating *in vitro* that HGF induces its anti-inflammatory effects by upregulating the production of IL-1ra [45]. Several authors speculate that HGF exerts its regenerative ability by induction of anti-inflammatory cytokines such as IL-1ra [46], [47]. HGF secretions were similar between WT and IL-1ra KO mice after hepatectomy, but as IL-1ra was not synthesized in the latter, HGF could not exert its regenerative ability.

Finally, reduced expression of transcription factors, immediate early genes and growth factors described here were associated with a delayed cell cycle transition of hepatocytes. The evaluation of cell cycle protein expression performed by analyzing cyclin D1 expression demonstrated that the start and the peak of expression was delayed in IL-1ra KO mice compared to WT mice. Because cyclin D1 regulates the G1/S cell cycle transition, the delay of cyclin D1 induction is likely also responsible for the delay and decrease in hepatocyte proliferation in IL-1ra KO mice.

The presented data indicate a potential mechanism for IL-1ra involvement in hepatocyte proliferation by promoting cell cycle transition from G1 to S phase. This could be mediated by IL-1ra released from hepatocytes to act upon the cell membrane IL-1 receptor [48]. Further, IL-1ra may act via an undefined intracellular mechanism to increase transcription of cell cycle proteins such as cyclin D1 in a similar way that it has been proposed for IL-1a [49] .

Anakinra, a non glycosylated recombinant human IL-1ra, was already used in various animal models of human diseases and also in several human clinical trials of rheumatoid arthritis with relative success [19], [50].

We evaluated the liver regeneration in WT mice treated with anakinra at 5mg/kg and at 50mg/kg after partial hepatectomy. Our results showed that hepatocyte proliferation was significantly higher only in animals treated with anakinra at 50mg/kg 24 h after partial hepatectomy. This finding can be explained by the fact that an efficient inhibition of IL-1 induced biological responses required injection of 100- to 1000-fold molar excess of IL-1ra. This might be due to the differential affinity of IL-1 and IL-1ra for IL-1 receptor [51], [52].

In our study, we showed that the treatment of WT mice with anakinra at 5mg/kg and at 50mg/kg after partial hepatectomy decreased levels of IL-6 but did not modify levels of IL-1β and of MCP-1 (fig. 7). These results are in line with two others studies. In a rat ischemia-reperfusion model, pre-treatment with IL-1ra gene delivery into the liver has been shown to decrease serum levels of IL-6 [17], [18]. In Shinoda et al reported an improvement of survival and a decrease of serum levels of IL-6 in a rat model of ALF treated by a bioartificial liver device containing transfected hepatocytes or by anakinra alone [14], [15].

In this study, we analyzed the levels of ALT on peripheral blood of WT mice treated with anakinra compared to mice without treatment. Our results showed that alanine aminotransferase increase at 4h after partial hepatectomy and returned to normal levels after 72h. The levels of alanine aminotransferase of WT mice without treatment was significantly higher at 24h after partial hepatectomy compared to mice treated with anakinra (5 or 50mg/kg). For treated mice, there was no difference for the protective effect of anakinra between 5 and 50mg/kg (Figure 7). These results indicate that the anti-inflammatory effect prevented further liver injury within the first 24 hours.

*In vitro*, we confirmed the proliferative effect of anakinra on isolated human hepatocytes. Primary human hepatocytes treated with anakinra showed a higher hepatocyte proliferation within the first 24 h compared to untreated cells (Figure 8A). This positive effect of IL-1ra may be related to its effects on cell proliferation as suggested by several studies using endothelial cells, pancreatic β-cells and hepatoyctes [4], [5], [53], [54], [55]. Indeed, Corsgrove et al. reported a pro-proliferative role of IL-1ra on rat hepatocyte proliferation when it was combined with TNF in vitro [56] . We analyzed the cell viability of primary human hepatocytes treated with anakinra and showed that the viability was similar for all culture conditions (with or without anakinra) at various time points (Figure 8B). These results suggest that the difference in cell proliferation between the human hepatocytes cultured with and without anakinra was not due to a difference of viability.

In conclusion, we provide evidence that liver regeneration is transiently impaired IL-1ra KO mice after 70% hepatectomy. Furthermore, decrease of pro-inflammatory cytokines (IL-6, MCP-1 and IL-1β), differences in the timing or level of secretion of cytokines (IL-6), of expression of cell cycle protein (cyclin D1) and of expression of genes (IGFBP-1, C/EBPβ, C-myc) that are involved in priming the hepatocyte for entry into the cell cycle or that regulate the G_1_/S checkpoint suggest that IL-1ra may contribute and modulate hepatocyte proliferation in the early phase of liver regeneration.
